# Dr. Thomas B. Harvey

**Published:** 1890-01

**Authors:** 


Dr. Thomas B. Harvey, of Indianapolis, Ind., died suddenly at his home on the evening of December 5, 1889, aged sixty-two years. Dr. Harvey was Professor of the Surgical and Clinical Diseases of Women in the Medical College of Indiana, and was engaged in Clinical teaching when he was stricken down with an apoplectic seizure. He was a picturesque character in the medical circles of the mid-West, an amiable man, and an able teacher—active in local, state, and National medical councils. He was present at the Newport meeting of the American Medical Association in June last, and also at the Evansville meeting of the Mississippi Valley Medical Association in September. His death at his post of duty was an ideal one for a physician, and a large circle of medical friends and acquaintances throughout the Union will join with his afflicted family in mourning his decease.

